# Versatile Supramolecular Complex for Targeted Antimicrobial Photodynamic Inactivation

**DOI:** 10.1021/acs.bioconjchem.2c00067

**Published:** 2022-03-10

**Authors:** Andrea Mussini, Eleonora Uriati, Cormac Hally, Santi Nonell, Paolo Bianchini, Alberto Diaspro, Stefano Pongolini, Pietro Delcanale, Stefania Abbruzzetti, Cristiano Viappiani

**Affiliations:** † Dipartimento di Scienze Matematiche, Fisiche e Informatiche, Università di Parma, Parco Area delle Scienze 7A, Parma 43124, Italy; ‡ Nanoscopy@Istituto Italiano di Tecnologia, Via Enrico Melen 83B, Genova 16152, Italy; § Institut Químic de Sarrià, Universitat Ramon Llull, Via Augusta 390, Barcelona 08017, Spain; ∥ DIFILAB, Dipartimento di Fisica, Università di Genova, Via Dodecaneso 33, Genova 16146, Italy; ⊥ Risk Analysis and Genomic Epidemiology, Istituto Zooprofilattico Sperimentale della Lombardia e dell’Emilia-Romagna, Strada dei Mercati, 13/A, Parma 43126, Italy

## Abstract

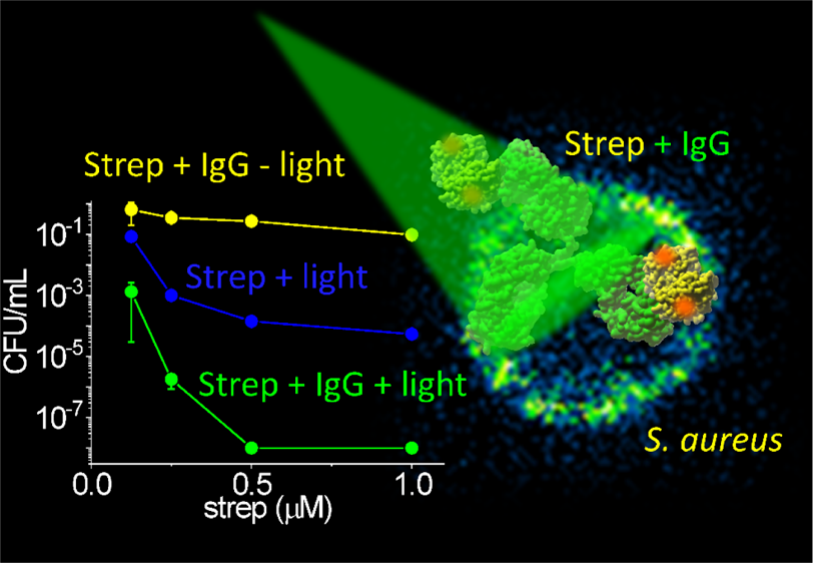

We report the development of a supramolecular structure endowed with photosensitizing properties and targeting capability for antimicrobial photodynamic inactivation. Our synthetic strategy uses the tetrameric bacterial protein streptavidin, labeled with the photosensitizer eosin, as the main building block. Biotinylated immunoglobulin G (IgG) from human serum, known to associate with *Staphylococcus aureus* protein A, was bound to the complex streptavidin–eosin. Fluorescence correlation spectroscopy and fluorescence microscopy demonstrate binding of the complex to *S. aureus*. Efficient photoinactivation is observed for *S. aureus* suspensions treated with IgG–streptavidin–eosin at concentrations higher than 0.5 μM and exposed to green light. The proposed strategy offers a flexible platform for targeting a variety of molecules and microbial species.

## Introduction

Antimicrobial resistance is emerging as one of the major health issues humanity will have to face in the next years. Misuse and abuse of antibiotics have led to the generation of widespread antimicrobial resistance.^1^ Close to 8.5 million deaths worldwide were caused by microbial infections in 2016, out of which around 700,000 have been associated with drug-resistant infections. In the lack of suitable actions, given the decreasing number of available effective antimicrobials, and the scarce rate of development of new replacements,^2^ a worst-case scenario envisions that in 2050, up to 10 million people could die annually due to antibiotic resistance.^3^ Research for alternative treatments, immune to resistance, is therefore of the highest importance.^4^

Antimicrobial photodynamic inactivation (PDI) is a promising approach in this direction.^5−8^ PDI relies on a photoactive molecule, named a photosensitizer (PS), capable of absorbing visible photons and undergoing intersystem crossing in high yield. The excess energy from the triplet state is used to generate highly reactive oxygen species (ROS), in many cases cytotoxic singlet oxygen (type II photo-process). Delivery of the PS has been accomplished through the use of several carriers.^7,9,10^

Proteins appear as useful carriers due to their intrinsic biocompatibility. We have previously reported water-soluble proteins as delivery systems for antimicrobial PDI, exploiting their non-covalent binding capability toward hydrophobic PS molecules.^11−17^ These passive carriers in general increase the PS solubility and bioavailability but are devoid of targeting capability. As a result, the PS is generally off-loaded to the membrane in the bacterial wall.^10^

A major step forward in PDI is the introduction of the capability of directing the photoactive compounds to specific molecular targets located on bacteria. Recent approaches have proposed the use of delivery systems, able to selectively address specific bacterial strains, through the conjugation of the PS to, for example, antibiotics or antibodies.^6,18−23^

A few examples of antibody–PS conjugates were proposed to target bacterial strains, mostly *Staphylococcus aureus* and methicillin-resistant *S. aureus* (MRSA). Protein A was previously exploited to enable selective photoinactivation of MRSA by the PS Sn-chlorin e6 linked to IgG. Higher efficiency was achieved with the conjugate PS-IgG than with the unconjugated PS at the same light energy dose and PS concentration.^21^ A further improvement was obtained through the use of an antibody raised against MRSA to make a conjugate between the antibody and Sn-chlorin e6, which proved capable of targeting several MRSA strains in all growth phases.^20^ Similarly, the near-infrared PS IRDye700DX was conjugated to a fully human mAb, specific for the invariantly expressed immune-dominant staphylococcal antigen A (IsaA).^24^

In this work, we propose to exploit protein A of *S. aureus* as a target for an immunoglobulin G (IgG)-associated photosensitizing supramolecular complex.^21,25^ Protein A is known to bind the Fc terminus of mammalian immunoglobulins in a nonimmune fashion, causing decoration of the staphylococcal surface with antibodies.^26^

In a modular approach, using streptavidin (strep) as a building block, we have chemically modified this protein by covalently linking the isothiocyanate derivative of the well-known PS eosin (eosin 5-isothiocyanate, EITC) to its Lys residues. In spite of the relatively low fluorescence yield (for eosin, the fluorescence yield in water is 0.24^27^), EITC has been used in the past to label avidin and applied in two-color confocal microscopy.^28^ However, the real potential of EITC for the current application derives from the fact that it is a good PS. When free in aqueous solutions, it has a quantum yield exceeding 0.5 for singlet oxygen generation,^29^ which makes it useful for photoconversion of electron-rich materials such as diaminobenzidine (DAB) or fluorescent dyes into highly electron-dense materials for high-resolution electron microscopy studies.^30^ EITC has also been exploited in photopolymerization reactions.^31^ The choice of eosin as a PS is motivated only by demonstration purposes and could be readily replaced by other PSs.

The resulting conjugate between EITC and strep (EITC–strep) was bound to biotinylated IgG, so that the resulting supramolecular assembly is endowed with photosensitizing properties and targeting capability. The above strategy could be specialized to different targets by replacing the IgG unit with suitable antibodies for the specific target and/or by conjugating strep to alternative photoactive molecules such as fluorescent probes, or PSs with improved singlet oxygen yield or better spectral properties than eosin.

## Results and Discussion

### Spectral Properties of EITC–Streptavidin

**Figure 1:**
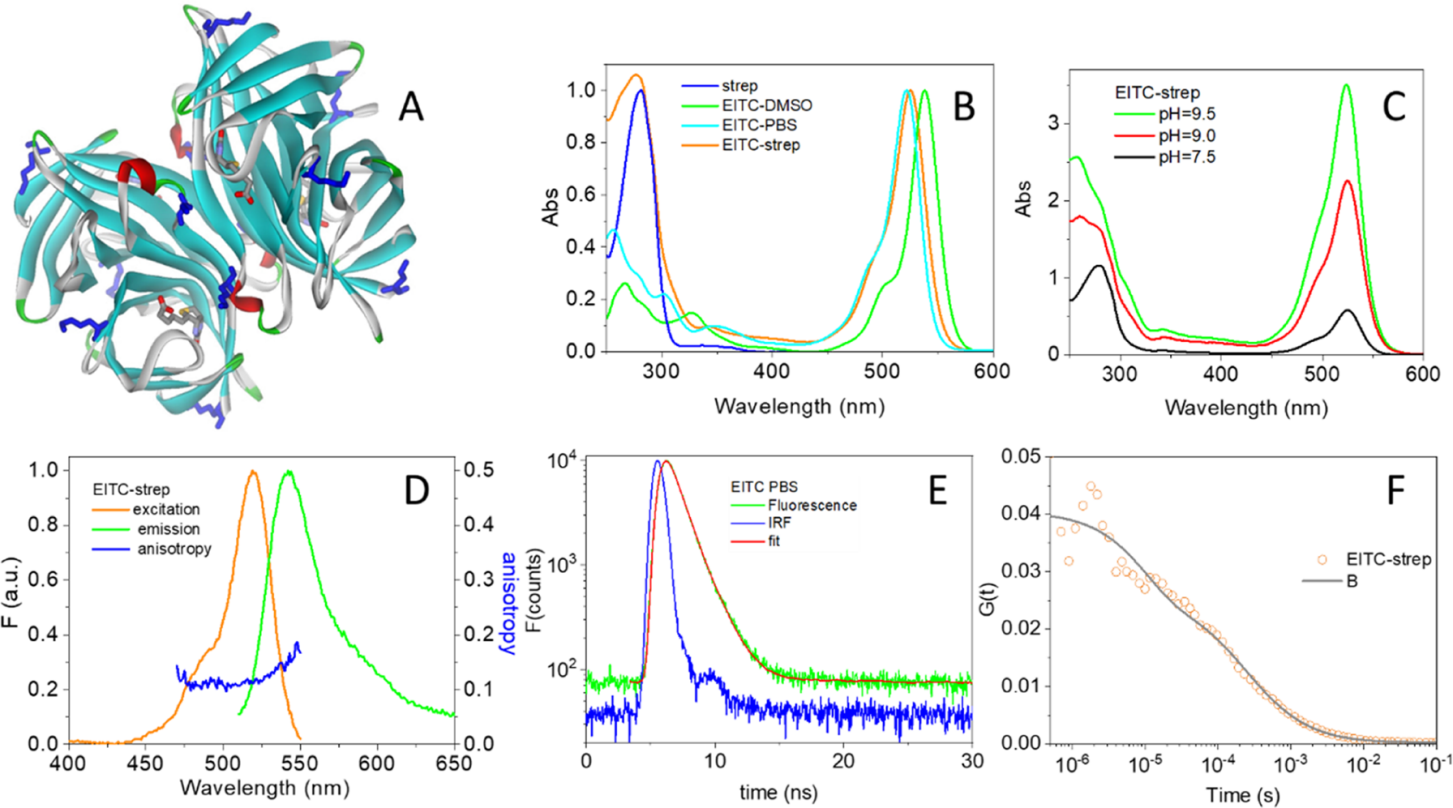
(A) Three-dimensional structure of strep from *Streptomyces avidinii* (PDB code 1n43, solid ribbon) bound to four biotin molecules. The Lys residues on strep are shown as blue sticks. The four biotin molecules bound to strep are shown as sticks. (B) Normalized absorption spectra of strep in PBS buffer at pH = 7.4 (blue), EITC in DMSO (green), EITC in PBS buffer (cyan), and the complex EITC–strep (at DOL ∼ 1) in PBS buffer at pH = 7.4 (orange). The absorption spectra were normalized at 280 nm (strep), 538 nm (EITC in DMSO), 521 nm (EITC in PBS), or 525 nm (EITC–strep in PBS). (C) Normalized absorption spectra of EITC–strep in PBS, pH = 7.4, purified after reaction at pH = 7.5 (black), pH = 9 (red), and pH = 9.5 (green). The spectra were normalized to the absorbance of the protein at 280 nm. (D) Normalized fluorescence excitation (orange, peak at 525 nm, emission collected at 580 nm) and emission (green, peak at 541 nm, excitation at 500 nm) for an EITC–strep solution in PBS buffer at pH = 7.4. Fluorescence excitation anisotropy is reported in blue (emission collected at 560 nm). (E) Fluorescence decay (TCSPC) for EITC in PBS buffer (green) compared with the IRF (blue). Excitation at 500 nm, detection at 600 nm. Best fit (red curve) was obtained with an exponential decay of lifetime τ_F_ = 1.1 ± 0.1. (F) Autocorrelation curve for EITC–streptavidin at DOL ∼ 1 (orange circles). Excitation was at 475 nm, detection at 550(20) nm in the cross-correlation mode. The solid curve is the result of a fit with a single diffusing species with diffusion coefficient *D* = 49 μm^2^/s and a triplet state with τ_T_ = 20 μs. In the reported experiment, about 25 molecules were present on an average in the confocal volume.

EITC shows an intense absorption in the visible with a maximum at 538 nm in DMSO, which shifts to 524 nm in PBS buffer. When bound to strep, the visible absorption band is found at 525 nm (Figure 1B). The extent of the reaction of EITC with strep is markedly affected by solution conditions, mostly by pH. Figure 1C shows the absorption spectra of the conjugates synthesized at selected pH values (7.5, 9, and 9.5), normalized to the absorbance of the protein at 280 nm. It is evident from visual inspection that the proportion of eosin-to-protein absorption, estimated from the ratio of the 525 to 280 nm absorbance (after correction for EITC absorbance at 280 nm), is increasing with pH. This is a clear indication of higher efficiency in the conjugation reaction at alkaline pH. From the extinction coefficients for strep monomers and for EITC, we calculated the degree of labeling (DOL) of strep monomers (the DOL is defined as the ratio [EITC]/[streptavidin monomers] and provides the average number of EITC bound to each strep monomer), which increases from 0.25 at pH = 7.5 to 1.0 at pH = 9 to reach 1.5 at pH = 9.5. This corresponds to having on an average about 1, 4, or 6 EITC molecules bound per streptavidin tetramer, respectively. It is worth observing that each strep monomer contains four potential reaction sites for EITC (Lys80, Lys121, Lys132, and the N terminal) (Lys residues displayed as blue sticks in Figure 1A), so the upper limit for the DOL in principle is 4.

**Figure 2:**
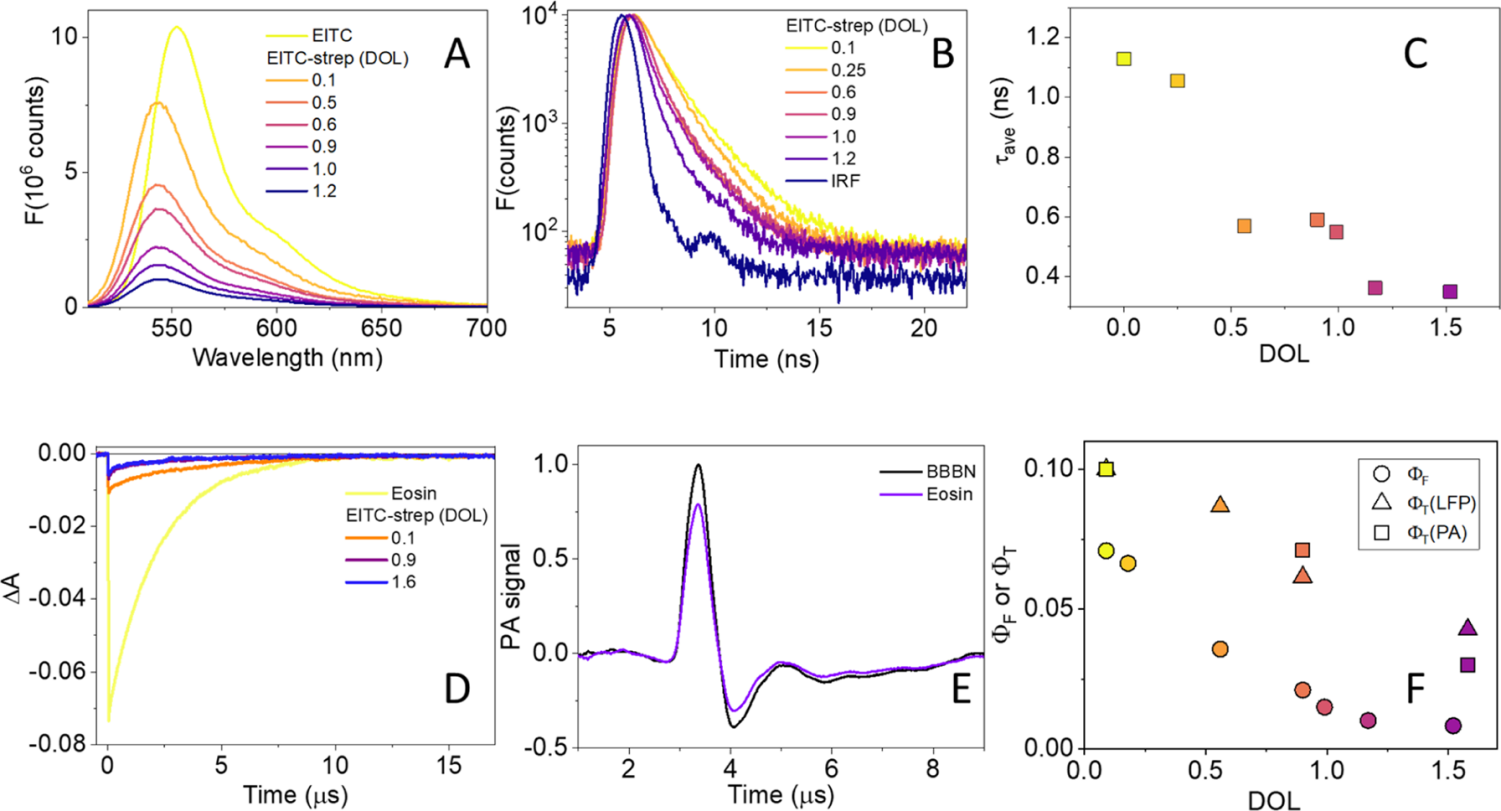
(A) Fluorescence emission by EITC (yellow) in PBS and EITC–strep at increasing DOL (orange to blue: 0.1, 0.5, 0.6, 0.9, 1.0, and 1.2), excitation at 500 nm. (B) Fluorescence emission decay (TCSPC) by EITC–strep at increasing DOL (yellow to purple: 0.1, 0.25, 0.6, 0.9, 1.0, and 1.2), excitation by a sub-ns LED at 500 nm (blue), and emission at 550 nm. (C) Singlet-state lifetime (squares) as a function of DOL determined from TCSPC data in panel B. (D) Triplet-state decay monitored at 500 nm for air-equilibrated PBS solutions of eosin (yellow), EITC–strep at DOL = 0.1 (orange), 0.9 (purple), and at DOL = 1.6 (dark blue). Excitation at 532 nm. (E) Normalized photoacoustic signals for EITC–strep (purple, DOL 0.1) and for the reference compound BBBN (black) at *T* = 20 °C. (F) Comparison between the fluorescence yield (circles) and triplet yield estimated with laser flash photolysis (triangles) and with time-resolved photoacoustics (squares) for EITC–strep as a function of DOL.

Figure 1D shows the emission from the conjugate (green curve), with a maximum at 541 nm, along with the fluorescence excitation spectrum (orange curve) that closely matches the absorption spectrum in Figure 1C. Fluorescence anisotropy of EITC in DMSO or PBS is negligible but becomes appreciable upon binding to streptavidin (blue curve in Figure 1D), indicating a strong reduction in rotational averaging of fluorescence polarization. This finding is consistent with the formation of the conjugate between the fluorophore and the protein. Figure 1E reports the fluorescence emission decay measured for EITC in PBS. The fluorescence lifetime of EITC in PBS is well described by an exponential decay with lifetime τ = 1.1 ± 0.1 ns. Figure 1F shows the autocorrelation function measured in the cross-correlation mode for EITC–strep (DOL ∼ 1). The curve is best described by a single diffusing species with diffusion coefficient *D* = 49 μm^2^/s plus a triplet-state contribution with τ_T_ ∼ 10 μs. The diffusion coefficient is consistent with literature values for the streptavidin tetramer, confirming conjugation of EITC to the protein.^32^ The low fluorescence emission by the conjugate (see Figure 2C) results in poor signal-to-noise ratio and prevents lowering the concentration below 50 nM. The triplet contribution shows that intersystem crossing occurs to an appreciable extent in the conjugate (vide infra).

Fluorescence emission intensity from EITC undergoes a progressive reduction upon binding to strep at increasing DOL, without any significant spectral change (Figure 2A). Fluorescence decays of EITC–strep conjugates reported in Figure 2B are best described by a sum of two exponential decays, with a fast component (whose lifetime is at the limit of the experimental resolution and is independent of the DOL) and a slower decay with a DOL-dependent lifetime. The relative amplitudes of the two transients are not affected by the DOL. The average lifetimes associated with the decays reported in Figure 2B are plotted in Figure 2C and show a general decrease at increasing DOL (about three-fold when the DOL is increased from 0.1 to 1.6). The change in singlet lifetime rules out homo-FRET between EITC molecules bound to the same strep tetramer as a source of the observed quenching.^33^ Using eosin as a reference in PBS, (Φ_F_ = 0.24),^27^ we have calculated the fluorescence yield for EITC–strep as a function of DOL (circles in Figure 2F). The trend observed for Φ_F_ is similar to the one obtained for the singlet lifetime, but the reduction is much higher (about 10-fold when the DOL is increased from 0.09 to 1.58).

The drop in fluorescence yield and singlet lifetime observed for EITC upon binding strep at higher DOL suggests that effective quenching of the excited single state occurs, leading to non-radiative de-excitation of increasing efficiency, which could be detrimental to triplet-state formation. We thus measured the triplet yield to assess whether the observed quenching has negative consequences on the singlet oxygen photosensitization. Figure 2D compares the triplet decay for eosin and EITC–strep at low (0.1) and high (0.9 and 1.6) DOL. The triplet decay was followed at 500 nm, where bleaching of the ground state is observed. The fluorescence emission upon photoexcitation leads to a spike on the short time scale for the air-equilibrated samples. The observed triplet lifetime for EITC in air-equilibrated PBS is 2.1 ± 0.1 μs, in keeping with the expected lifetime for a triplet quenched by molecular oxygen ([O_2_] ∼ 0.2 mM for air-equilibrated PBS buffer). When EITC is bound to strep in air-equilibrated solutions, the triplet lifetime is a bit (2- to 3-fold) longer, indicating that the triplet state is somewhat protected from molecular oxygen present in solution. When solutions are saturated with nitrogen, the lifetime of the triplet state of EITC in PBS becomes comparable when free or bound to streptavidin (∼1.5 ms, data not shown). Using eosin as a reference (Φ_T_ = 0.7, average of value in Table 1), we estimated that Φ_T_ for EITC–strep in air-equilibrated PBS buffer (Φ_T_ ∼ 0.09) is smaller than the corresponding value for EITC in PBS (Φ_T_ = 0.49). The triplet yield is affected by the DOL (squares in Figure 2F) with a three-fold decrease when the DOL increases from 0.1 to 1.6.

**Table 1:** Photophysical Parameters of Eosin and Its Derivatives

	Φ_F_	τ_F_ (ns)	Φ_T_	τ_T_ (μs)
eosin	0.2^a^^,^^36,37^	1.21^c^^,^^27^	0.64^a^^,^^38^	1.7 ± 0.1^c^^,^^f^^,^^h^
	0.24^c^^,^^27^	1.2 ± 0.1^c^^,^^h^	0.71^a^^,^^39^	1440 ± 50^c^^,^^g^^,^^h^
			0.8^a^^,^^37^	
EITC	0.18^c^^,^^h^	1.1 ± 0.1^c^^,^^h^	0.49^c^^,^^e^^,^^h^	2.1 ± 0.1^c^^,^^f^^,^^h^
	0.58^b^^,^^h^	3.1 ± 0.1^b^^,^^h^	0.06^b^^,^^e^^,^^h^	2.450.1^b^^,^^f^^,^^h^
EITC–strep^i^	0.071 (0.1)^c^^,^^h^	1.2 ± 0.1 (0.1)^c^^,^^d^^,^^h^	0.10 (0.1)^c^^,^^f^^,^^h^	5.1 ± 0.1 (0.1)^c^^,^^f^^,^^h^
	0.008 (1.5)^c^^,^^h^	0.3 ± 0.1 (1.5)^c^^,^^d^^,^^h^	0.04 (1.6)^c^^,^^f^^,^^h^	4.5 ± 0.1 (1.5)^c^^,^^f^^,^^h^
				1140 ± 50 (1.5)^c^^,^^g^^,^^h^

a Water.

b DMSO.

c PBS buffer.

d Average lifetime τ_av_ = (α_1_τ_1_ + α_2_τ_2_)/(α_1_ + α_2_).

e Calculated using the value of 0.8 for eosin in water.

f Air equilibrated solutions.

g Nitrogen-saturated solutions.

h This work.

i Values in brackets are DOL.

Formation of the triplet state in EITC–strep was also evident in the short time scale of the FCS trace (Figure 1F).

The triplet yield was also determined by means of time-resolved photoacoustics in order to get an independent estimate. Figure 2E shows representative normalized photoacoustic signals for EITC–strep (purple, DOL 0.09) and for the reference compound BBBN (black) at *T* = 20 °C. The photoacoustic signals for eosin, EITC, and EITC–strep were best described by double-exponential decays. A prompt decay (i.e., with lifetime below the experimental resolution), associated with singlet-state relaxation, is followed by a second, slower phase, associated with triplet-state and singlet oxygen decay. The close values of the triplet state and singlet oxygen lifetimes prevent separation of the transients, and an intermediate value for the lifetime is obtained (2.7 μs for eosin and 5.2 μs for EITC–strep at DOL 0.1 and *T* = 20 °C), with an amplitude reflecting the energy content of the overall decay.^34^

Each transient is characterized by heat release *Q*_i_ and volume change Δ*V*_i_. We concentrate on the heat released in the triplet (and singlet oxygen) decay (*Q*_2_) because this provides us with a direct estimate of the triplet yield. *Q*_2_ can be written as *Q*_2_ = Φ_T_*E*_T_. Since the energy of eosin triplet state can be estimated as *E*_T_ = 42 kcal/mol from the room-temperature phosphorescence (peaked at ∼680 nm),^35^ Φ_T_ can be readily derived as Φ_T_ = *Q*_2_/*E*_T_. For eosin, we estimate *Q*_2_ = 28 ± 2 kcal/mol that affords Φ_T_ = 0.67 ± 0.05. The values of Φ_T_ for EITC–strep at DOL of 0.1, 0.9, and 1.6 are reported as the squares in Figure 2F.

The photophysical parameters of eosin, EITC, and EITC–strep at selected DOL values are summarized in Table 1.

### EITC Labeling Does Not Affect Biotin Binding Sites on Streptavidin

**Figure 3:**
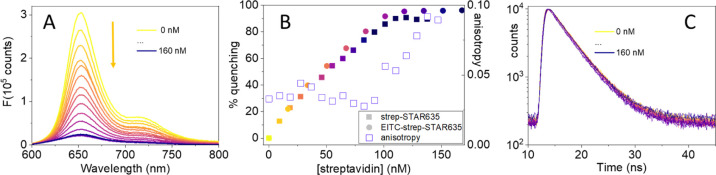
(A) Fluorescence emission by biotinylated STAR635 (100 nM) as a function of streptavidin concentration from 0 (top) to 160 nM (bottom). The arrow indicates the direction of streptavidin concentration increase. (B) Fluorescence emission intensity (integrated area) reduction by biotinylated STAR 635 (100 nM) upon increasing streptavidin (circles) or EITC–strep (squares) concentration. The DOL for the EITC–strep complex reported in the plot was 1, but similar results are obtained for all DOL. The concentration reported in the plot refers to streptavidin monomers. The open squares report the change in fluorescence anisotropy for the titration with streptavidin. (C) Fluorescence decay for biotinylated STAR635 (100 nM) as a function of streptavidin concentration.

We have next estimated the consequences of EITC conjugation on the functional properties of streptavidin by determining the number of available biotin binding sites on the streptavidin tetramer. For this purpose, we took the advantage of the quenching of fluorescence emission by the biotinylated fluorescent dye STAR635 upon binding to streptavidin. As shown in Figure 3A, the STAR635 fluorescence emission intensity progressively decreases upon binding to streptavidin. From the fluorescence emission, we calculated the percent emission reduction and plotted this quantity as a function of strep concentration (Figure 3B). The emission reduction continues until all STAR635 molecules have bound strep (circles). The fluorescence intensity quenching is accompanied by a small decrease in fluorescence lifetime (Figure 3C).^33^ The linear increase in Figure 3B indicates that binding of biotin-STAR635 is stoichiometric. The strep concentration at which the linear increase saturates provides a means to evaluate the concentration of the available binding sites. In particular, the ratio between the biotin-STAR635 and the EITC–strep concentrations is a direct estimate of the fraction of the available binding sites. Very similar results were obtained when biotin-STAR635 is titrated with EITC–strep (squares in Figure 3B), an indication that all biotin binding sites are available in this conjugate. Importantly, this property is observed for all EITC–strep conjugates, regardless of their DOL.

### Binding of Biotinylated IgG to EITC–Streptavidin

**Figure 4:**
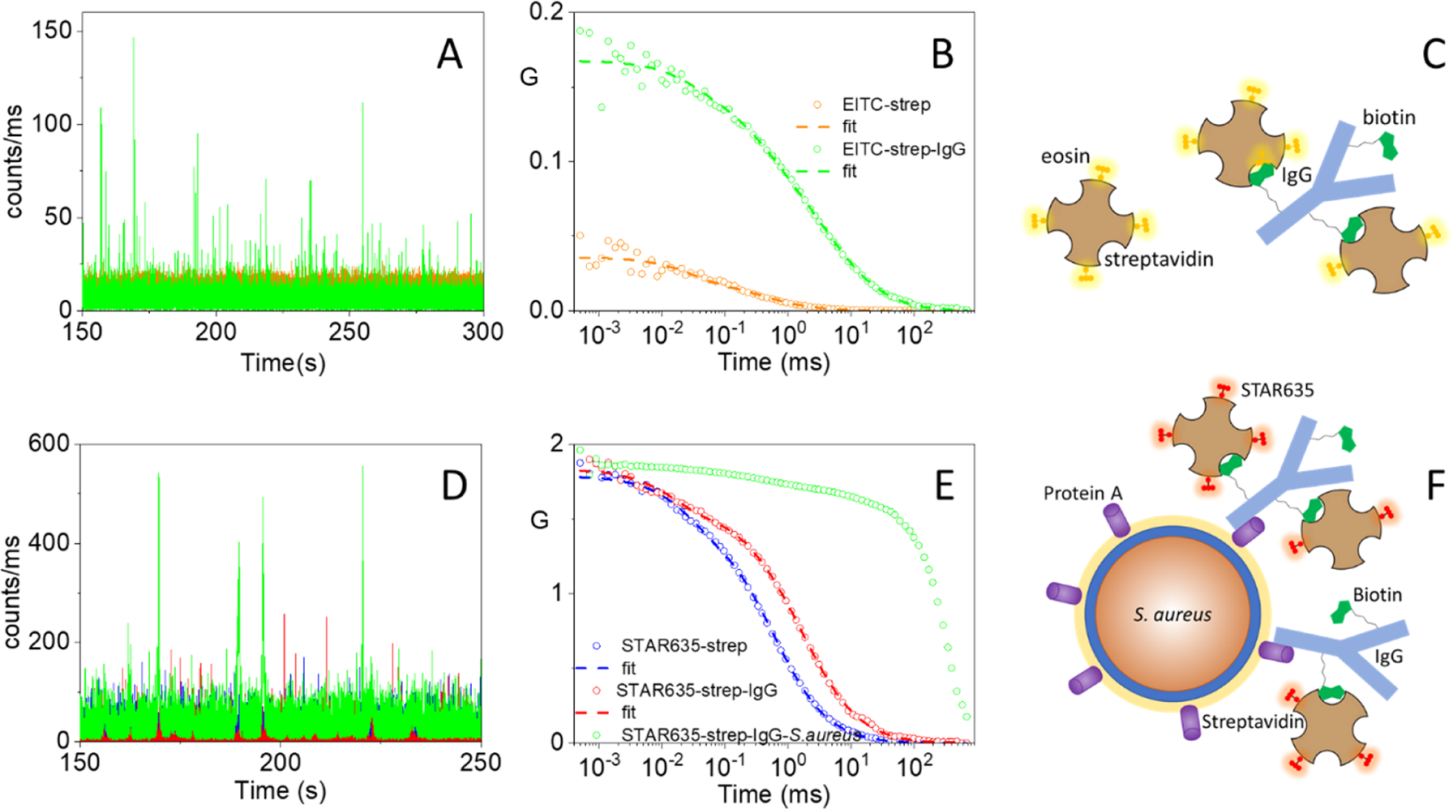
(A) Photon count traces for EITC–strep 60 nM (orange trace) and for EITC–strep incubated with 32 nM IgG (green trace). Photon binning is 1 ms. Excitation at 475 nm, detection at 550/20 nm. (B) Cross-correlation curves for EITC–streptavidin in PBS (orange circles, 60 nM) and in the presence of 32 nM IgG (green circles). pH = 7.4. The dashed lines are the result of the fitting with a model comprising a triplet state and one (orange) or two (green) diffusing species. (C) Cartoon representing interactions between EITC–streptavidin and biotinylated IgG. (D) Photon count traces (portion of 100 s) for STAR635-strep 5 nM (blue trace, barely visible in the background) and for STAR635-strep incubated with 25 nM IgG (red trace). The green trace reports the signal measured after incubation of the above solution with a suspension of *S. aureus*. Photon binning is 1 ms. Excitation at 635 nm, detection at 650 nm. (E) Autocorrelation functions for signals in Figure 4D on a 10 min time window. The dashed lines are the result of the best fits to the autocorrelation functions for STAR635-strep (blue) and for STAR635-strep–IgG (red). (F) Cartoon showing a schematic of the interactions between the supramolecular complexes and protein A on *S. aureus*.

The fully functional biotin binding sites on EITC–strep (Figure 3B) warrant self-assembly of a supramolecular complex with biotinylated IgG. Binding of EITC–strep to biotinylated IgG does not lead to major changes in the spectral features of EITC–strep (data not shown). However, binding of biotinylated IgG to EITC–strep is easily detected using fluorescence correlation spectroscopy (FCS). Figure 4A shows a 150s-portion of the photon count trace for EITC–strep (orange trace) at 60 nM (tetramer concentration), where it is possible to distinguish the low-intensity (due to the weak fluorescence emission by EITC–strep) fluctuations generated by protein diffusion. The associated autocorrelation curve for EITC–streptavidin (orange trace in Figure 4B) is best described by a single diffusing species with diffusion coefficient *D* ∼50 μm^2^/s, in agreement with literature values,^32^ and a triplet decay with lifetime around 10 μs. Upon addition of increasing concentrations of biotinylated IgG, the cross-correlation curve becomes progressively slower due to binding between IgG and EITC–strep, as shown in Figure 4B for [IgG] = 32 nM (green circles). The time course is now best described by two diffusive species, one with *D* = 50 μm^2^/s, corresponding to EITC–strep, and one with *D* ∼5 μm^2^/s. The latter species is identified as larger size complexes formed through the multiple interaction points between EITC–strep (with 4 equiv biotin binding sites) and biotinylated IgG (with ∼10 biotins per protein), as suggested in the cartoon in Figure 4C. Recruiting of several EITC–strep molecules in each complex results in spikes of large intensity (green trace in Figure 4A) and decreases the average number *N* of diffusing molecules in solution within the confocal volume (Figure 4B, where *G*(0) = 1/*N*). For the curves in Figure 4B, the value of *N* has decreased from 20 (orange) to 5 (green), an indication that complexes involving about four EITC–strep conjugates are formed. It is worth observing that although these experiments do not allow us to determine quantitatively the affinity of the biotinylated IgG for the modified strep, they suggest that the dissociation constant cannot exceed a few nM.

### Full Construct Binds *S. aureus*

Although fully functional in terms of photophysical properties, fluorescence emission from the IgG–EITC–strep complex is quite weak. To assess binding of the full construct to *S. aureus* through FCS, we thus replaced EITC with a brighter fluorophore emitting in the red (STAR635). Bacteria were first incubated for 30 min with IgG, so that the antibody binds protein A on the bacterial wall, then exposed cells to STAR635-strep for an additional hour, and their time trace collected under 635 nm excitation. Figure 4D compares the photon count traces for 5 nM STAR635-strep (blue trace) and for 5 nM STAR635-strep incubated with 25 nM IgG (red trace). The green trace in Figure 4D reports the signal measured with a suspension of *S. aureus* incubated for 30 min with biotinylated IgG (binding to protein A) and then exposed for 1 h to STAR635-strep (binding to biotinylated IgG). Several very high intensity spikes appear in the green time trace due to slowly diffusing bacteria, loaded with multiple copies of the fluorophore. The resulting autocorrelation curves are reported in Figure 4E where the signals have been normalized to that of STAR635-strep to allow easier comparison. The autocorrelation curve for STAR635-strep (blue curve) is best described by a single diffusing species with *D* = 45 μm^2^/s and a triplet term with a lifetime of 30 μs. For STAR635-strep incubated with 25 nM IgG (red curve), we observe a minor amplitude (∼20%) diffusing species with *D* ∼45 μm^2^/s, corresponding to STAR635-strep, and a dominant (∼80%) species with *D* = 12 μm^2^/s, which corresponds to large-scale supramolecular assemblies, comprising several STAR635 and IgG molecules. In the presence of bacteria, the autocorrelation curve undergoes a dramatic change, best described by a dominant diffusing species with lifetime in the order of the hundreds of ms and a small amplitude component that accounts for residual unbound protein molecules. Unfortunately, it is difficult to estimate the diffusion coefficient of the slow diffusing species, possibly due to the heterogeneity in size induced by bacterial aggregates. Figure 4F offers a cartoon view of the interaction between the supramolecular complexes and protein A on *S. aureus*.

**Figure 5:**
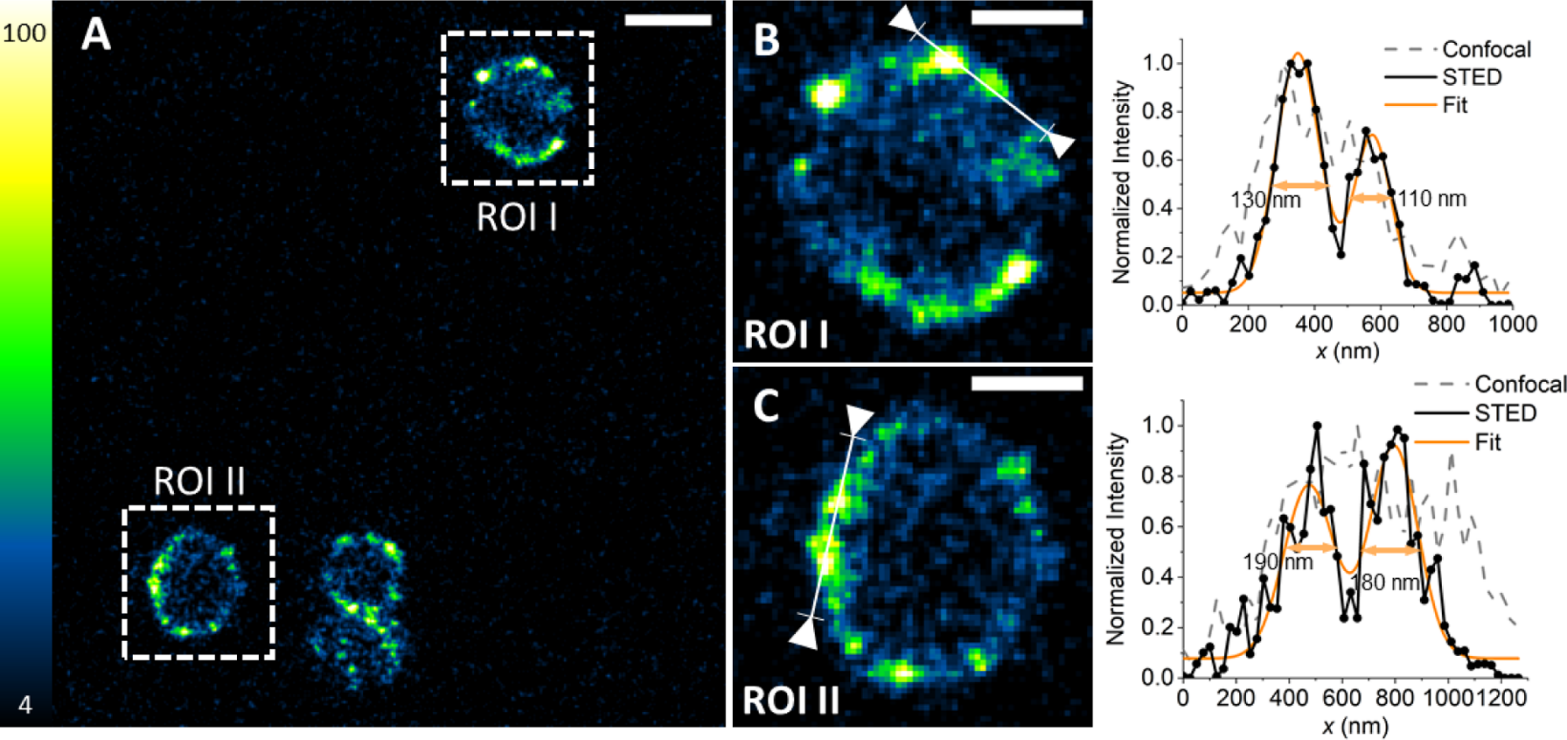
Labeling of *S. aureus* with Chromeo488-strep–IgG. Bacteria were incubated with IgG and Chromeo488-strep (0.5 and 1 μM, respectively). (A) STED image collected under excitation at 488 nm and detection 495–550 nm using a depletion beam at 592 nm. The fluorescence signal is distributed on the bacterial wall. Scale bar 1 μm. (B,C) Magnified regions from the STED image. Scale bars 500 nm. Normalized intensity profiles measured across interesting domains on the bacterial wall are shown to the right of the corresponding magnified images. In the same intensity plots, the confocal profile is shown in comparison to the STED one (acquisition taken in a second sequential frame). A multi-peak fit (orange line) has been used to assess the dimension of the domains. Estimated widths are reported in each plot.

Binding of the supramolecular structure to protein A of *S. aureus* can be further appreciated by means of fluorescence microscopy. Figure 5A shows a STED image of *S. aureus* cells incubated with biotinylated IgG and then exposed to strep labeled with chromeo488. As expected, fluorescence emission is observed from the bacterial wall, where protein A is located. Simple visual inspection reveals that fluorescence is unevenly distributed on the wall and arises from well separate spots. Uneven distribution of protein A on the bacterial wall has been reported and discussed in relation to cellular division.^26,40^

Figure 5B,C offers expanded views of ROI I and II. The intensity profiles along the lines indicated in ROI I and ROI II are reported in the right parts of panels B and C for the confocal (dotted lines) and the STED (solid lines) images. STED and confocal images have been taken in frame sequence. From the plots, it is possible to appreciate that fluorescent spots are well separated in the STED images and have size on the order of 100–200 nm. These images unequivocally show that the supramolecular structure targets *S. aureus*’s wall.

**Figure 6:**
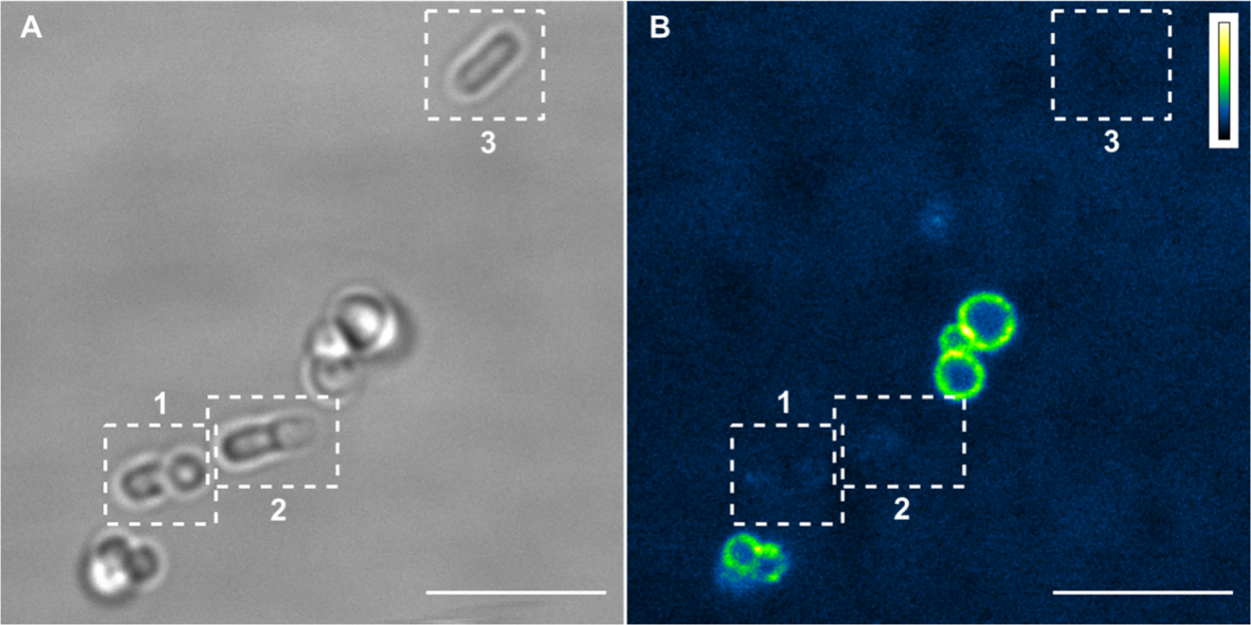
Selective labeling of *S. aureus* with chromeo488-strep–IgG in a mixed culture with *E. coli*. (A) Transmitted light image of bacterial cells showing the different morphology of *S. aureus* (spherical) and *E. coli* (rod-shaped). The latter ones are highlighted in boxes 1, 2, and 3. Scale bar 5 μm. (B) Confocal image collected under excitation at 488 nm and detection 495–550 nm. Scale bar 5 μm. Interestingly, the fluorescence signal is absent on *E. coli* walls (boxes 1, 2, and 3 in B).

Besides having targeting capability, the supramolecular structure is endowed with strain selectivity toward bacteria expressing protein A, as exemplified in Figure 6. *E. coli* (devoid of protein A) and *S. aureus* (expressing protein A) cultures were grown and mixed. The mixed culture was then incubated with biotinylated IgG for 30 min and exposed to chromeo488-strep for additional 30 min. Confocal microscopy (Figure 6B) shows that fluorescence from the construct is detectable only from *S. aureus*, whereas *E. coli* cells, which can be easily identified in transmission (Figure 6A) and are indicated by boxes 1, 2, and 3, are devoid of any fluorescence emission.

### Photodynamic Inactivation of *S. aureus* with IgG–EITC–Strep

In order to assess the photoinactivation efficiency of the supramolecular complex IgG–EITC–strep, we performed in vitro photoinactivation assays on *S. aureus* suspensions.

*S. aureus* suspensions were incubated with biotinylated IgG (100 nM) for 30 min to allow for binding of the antibody to protein A. EITC–strep was then added to the suspensions at increasing concentrations from 125 nM to 1 μM (tetramer concentration) and further incubated for 30 min to allow binding to protein A-linked biotinylated IgG. The treated bacteria were exposed to green light at increasing exposure times, corresponding to light fluences of 0 (dark), 10, 20, and 50 J/cm^2^.

**Figure 7:**
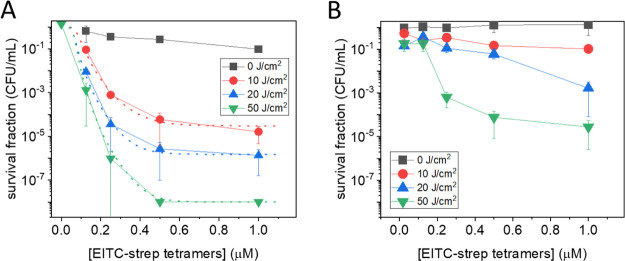
Light fluence and PS concentration-dependent PDI of *S. aureus*. (A) Plot of the cfu/mL survival fraction as a function of EITC–strep tetramers concentration (0.125, 0.25, 0.5, and 1 μM) in the solution at fluences of 0 (dark control; black), 10 (red), 20 (blue), and 50 (green) J/cm^2^. Biotinylated IgG was 100 nM. The dotted lines are the result of the fit to a Hill equation and are intended to provide an estimate of the trend when extrapolated to [EITC–strep] = 0.^41^ (B) Control experiment for which *S. aureus* was not pretreated with biotinylated IgG. The remaining conditions were as in A.

Figure 7A shows the fluence and EITC–strep concentration dependence for photoinactivation of *S. aureus* suspensions treated with IgG–EITC–strep. From the plot, it is evident that the reduction in cfu/mL correlates with both PS concentration and light fluence. A saturating reduction of 8 log at a concentration of EITC–strep tetramers of 0.5 μM and a fluence of 50 J/cm^2^ was observed.

Control experiments showed that no photoinactivation is obtained with an illumination of untreated bacteria at the highest light fluence used in this work (green triangle at [EITC–strep] = 0 in Figure 7A). The dotted lines in Figure 7A are the best fits with a dose–response model^41^ for the different light fluences investigated. It can be easily appreciated that they all extrapolate to a survival fraction of one at [EITC–strep] = 0, supporting the absence of light-only photoinactivation. Incidentally, this also rules out possible heating effects induced by illumination with the LED lamp.

To assess the role of the specific interaction between IgG and protein A in driving the photosensitized photoinactivation of *S. aureus*, we have performed a control photoinactivation experiment on bacteria treated with EITC–strep without pretreatment with biotinylated IgG. Figure 7B reports the results obtained in these control experiments. A residual photoinactivation is observed in response to EITC–strep treatment, indicating that bacteria show some minor interaction with the photosensitizing conjugate. At the concentration of EITC–strep tetramers of 0.5 μM and the fluence of 50 J/cm^2^, a 4 log reduction in cfu/mL was observed. This can be compared with the 8 log reduction obtained in the presence of biotinylated IgG. The remarkable improvement introduced by the use of the antibody demonstrates the advantage of the specific interaction between protein A and IgG in driving the photoinactivation.

The residual activity of EITC–strep is likely related to the interaction of strep with endogenous biotin molecules on the cell wall of *S. aureus*, as previously reported.^42^

## Conclusions

In this work, we have developed a versatile supramolecular construct around tetrameric protein streptavidin by introducing eosin isothiocyanate through covalent labeling of Lys residues and exploiting the biotin binding sites to bind a biotinylated antibody (IgG). The full conjugate is photoactive, binds *S. aureus*, and induces efficient photoinactivation of this bacterial strain at nM concentrations.

The modular approach, based on streptavidin as the main building block, enabled the replacement of the photoactive species eosin with other photoactive species such as bright fluorophores, in order to track molecular interactions and cellular recognition. We can also envision that different photosensitizing molecules can replace eosin in order to improve spectral properties. Thanks to the synthetic approach, the targeting part of the supramolecular construct can be specialized to other molecular targets, by employing a biotinylated antibody or another molecular species that is recognized and bound by a receptor on the surface of the targeted cell.

## Materials and Methods

IgG from normal human serum, streptavidin (strep) from *Streptomyces avidinii* (salt-free, lyophilized powder, see Figure 1A for the three-dimensional structure of the complex between the tetramer and biotin, PDB code 1n43), and eosin 5-isothiocyanate were from Sigma-Aldrich. The Biotin Protein Labeling Kit was from Biotium, Inc. (Fremont, CA, USA). Biotinylated STAR635 and STAR635 labeled streptavidin were from Abberior GmbH (Göttingen, Germany). Strep labeled with Chromeo488 was from Active Motif, Inc.

Concentrations were estimated from the molar extinction coefficients of the compounds: ε(280 nm) = 41,326 cm^–1^ M^–1^ for streptavidin monomers, ε(538 nm) = 95,000 cm^–1^ M^–1^ and ε(280 nm) = 26,766 cm^–1^ M^–1^ for EITC (in DMSO), and ε(280 nm) = 210,000 cm^–1^ M^–1^ for IgG. The strep concentration in EITC–strep complexes was estimated from the absorbance at 280 nm after correcting for the EITC contribution at this wavelength. It is assumed that the molar extinction coefficient of the visible absorption band of EITC is not affected by binding to strep.

PD Minitrap G-25 was from Cytiva (Marlborough, MA, USA).

### IgG Biotinylation

Biotinylation of IgG was performed using a biotin protein labeling kit based on a succinimidyl ester biotin derivative^43^ (Biotin SE Protein Labeling Kit, Biotium, Inc., Fremont, CA, USA). The overall yield in protein after purification exceeded 50%.

### EITC–Streptavidin Conjugate

The conjugate between EITC and strep was prepared using a standard protocol. EITC was dissolved in DMSO at 9 mM. The concentrated EITC solution in DMSO (125 μL) was added to a 100 μM (monomer concentration) strep solution (1 mL) in 0.1 M sodium carbonate buffer (pH > 9) or in PBS (pH < 9) to a final EITC concentration of 900 μM. The solution was stirred at 4 °C overnight. The protein conjugate was purified using a Sephadex G25, PD-10 column equilibrated with PBS buffer at pH = 7.4.

Each strep monomer contains four potential reaction sites for EITC (Lys80, Lys121, Lys132, and the N terminal) (Lys residues displayed as blue sticks in Figure 1A). This implies that the DOL of strep monomers, defined as the ratio [EITC]/[strep monomers] and providing the average number of EITC bond to each strep monomer, has a theoretical upper limit of 4. Under these conditions, the number of EITC per tetramer would reach the upper limit of 16. In our experiments, the DOL never exceeded 1.5 corresponding to six EITC molecules per tetramer. The labeling reaction was performed at pH values between 7.5 and 10.5, obtaining the highest yield for the most alkaline conditions.

To rule out the formation of non-covalent adducts between EITC and strep, control experiments were conducted incubating strep with the parent compound eosin under the same conditions (pH, concentration, and incubation time) used for labeling the protein with EITC. When the solution containing strep and eosin was applied to the PD-10 column, the dye was trapped in the column and the collected fractions contained only strep, as judged by the absorption spectra. This confirms the lack of a strong non-specific, non-covalent interaction between the dye and strep.

Further, mass spectrometry showed that the mass difference between unlabeled and EITC-labeled strep monomers is consistent with the mass of bound EITC (see the Supporting Information, Figure S1).

### Photoinactivation of *S. aureus* Suspensions

Vegetative *S. aureus* ATCC 25923 cells were grown in sterile tryptic soy broth at 37 °C until an optical density of 0.4 at 600 nm corresponding to an initial concentration of bacteria of 10^7^ cfu/mL. Cell suspensions were then washed three times in PBS by means of centrifugation and resuspension. In one type of experiment, the cells were first incubated in the dark with the biotinylated IgG at 100 nM for 30 min at room temperature and then incubated for 30 more minutes with EITC–strep (at strep tetramer concentrations of 0.125, 0.25, 0.5, and 1 μM; DOL = 1, vide infra). In a control experiment, the cells were incubated for 30 min with EITC–strep (at strep tetramer concentrations of 0.125, 0.25, 0.5, and 1 μM; DOL = 1, vide infra) in the absence of the antibody.

Photoinactivation experiments were performed as previously described.^12^ Suspensions were placed in 96-well plates and irradiated with green light using a LED light source (SORISA Photocare) for which the green output at 521 ± 19 nm (27.5 mW/cm^2^) was selected.

Irradiation was performed for 6, 12, or 30 min (corresponding to light fluences of 10, 20, and 50 J/cm^2^, respectively). Irradiation only (without exposure to the photoactive compounds) leads to no appreciable effects on bacterial growth.

Suspensions were then serially diluted until 10^–6^ times the original concentration and then plated on tryptic soy agar plates. Colony forming units (cfus) were counted after 24 h incubation in the dark at 37 °C to calculate the survival fraction. Three independent assays were conducted, with six replicates within each assay. Survival fractions are expressed as means ± standard deviation.

### Spectroscopy

Absorption spectra were collected using a Jasco V-650 (Jasco Europe) spectrophotometer. Steady-state fluorescence excitation, emission, and anisotropy spectra were measured with a SF5 spectrofluorometer (Edinburgh Instruments Ltd., Livingston, UK). Fluorescence decays were recorded by a FLS920 time-correlated single-photon counting system (TCSPC) (Edinburgh Instruments Ltd., Livingston, UK) with pulsed LED excitation at 500 or 600 nm operated at 5 MHz repetition rate. The quality of the fitting was evaluated through the value of the reduced χ^2^ (∼1.0–1.5) and visual inspection of residuals and the autocorrelation of residuals.

Fluorescence quantum yields were determined with a comparative method^44^ using eosin in aqueous solution as a reference compound (Φ_F_ = 0.24).^27^

All experiments were performed at 20 °C.

#### Laser Flash Photolysis

Triplet-state decays of EITC and EITC–strep were monitored at 500 nm after photoexcitation with the second harmonic (532 nm) of a nanosecond Nd/YAG laser (Surelite I-10, Continuum, San Jose, CA, USA) using a previously described setup.^45,46^ Triplet quantum yields were estimated from laser flash photolysis using eosin in aqueous solution as a reference compound (Φ_T_ = 0.8^37^).

#### Time-Resolved Photoacoustics

The photoacoustic setup was described previously.^46^ Photoexcitation was achieved by the second harmonic (532 nm) of a nanosecond Nd/YAG laser (Surelite I-10, Continuum, San Jose, CA, USA). The beam was shaped with a 280 μm slit, and the pressure wave was detected by a piezoelectric transducer (Panametrics V-103) and amplified before being fed to a digital scope (LeCroy 9370). The temperature was controlled by a FLASH 100 sample holder (Quantum Northwest, Inc.). Experiments were conducted at temperatures between 5 and 30 °C.

The thermodynamic and kinetic information was retrieved from photoacoustic waveforms as described.^47^ The sample waveform is assumed to be convolution of a reference waveform obtained with a compound releasing all of the absorbed energy as heat within a few nanoseconds and a sum of exponential decay functions.^48^
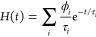


In this equation, φ_*i*_ is the pre-exponential factor of the transient with lifetime τ_*i*_. A dedicated deconvolution analysis software (Sound Analysis, Quantum Northwest, Inc.) was used to retrieve φ_*i*_ and τ_*i*_.^49,50^ Each pre-exponential factor φ_*i*_ contains contributions from heat released (*Q*_*i*_) and structural volume changes (Δ*V*_*i*_). We have then calculated *Q*_*i*_ (from the intercept) and Δ*V*_*i*_ (from the slope) for each of the detected transients, taking advantage of the linear relation between φ_*i*_*E*_λ_ and *C*_p_ρ/β.^47,51^
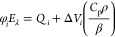


#### Fluorescence Correlation Spectroscopy

FCS experiments were performed using a Microtime 200 system from PicoQuant based on an inverted confocal microscope (Olympus IX71) and equipped with two SPADs (single photon avalanche diodes) used in the cross-correlation mode. The excitation was achieved by a 475 or a 635 nm picosecond diode laser operated at 20 MHz. Fluorescence emission by EITC–strep was collected through a band-pass filter (555/20 nm) and split with a 50/50 splitter between the two detection channels. Fluorescence from STAR635 was collected through a band-pass filter (670/20 nm).

### Microscopy

#### Sample Preparation for Imaging

Vegetative *S. aureus* ATCC 25923 cells were grown in 5 mL of sterile LB broth at 37 °C overnight. A volume of 1 mL of this cell suspension was then washed four times in PBS buffer by means of centrifugation (10 min at 4000 rpm) and suspended again. *S. aureus* washed cells were subsequently diluted six times in a final volume of 1 mL. A sample in a final volume of 500 μL of bacterial cells was prepared with a 0.5 μM concentration of IgG, undergoing an incubation time of 30 min at 37 °C and a 10 min centrifugation cycle at 4000 rpm. The supernatant was discarded, and bacterial cells were suspended in 500 μL of PBS buffer with a 1 μM concentration of Chromeo488-Strep, followed by an incubation time of 30 min at 37 °C and a 10 min centrifugation cycle at 4000 rpm. The supernatant was discarded, and the bacterial cells were suspended in 500 μL of PBS buffer. Imaging measurements were performed using a live-cell imaging culture chamber (compatible with 18 mm round coverslips). Coverslips (18 mm) were prepared with poly-d-lysine and subsequently used after a PBS buffer wash. The prepared bacterial cell stock of 500 μL was diluted 10 times in the imaging chamber, where 30 μL of the stock solution was added to 270 μL of PBS buffer for a total volume of 300 μL. In the mixed culture experiment, vegetative *E. coli* cells BL21 were used.

#### Confocal Microscopy and STED Nanoscopy

The microscope used for the confocal and super-resolved measurements was a Leica TCS SP5 STED equipped with a CW laser for excitation and a depletion line at 592 nm. In all the experiments, the excitation wavelength was 488 nm. The detector used for image acquisition was an HyD detector with increased sensitivity in a spectral window of 495–550 nm. The filters used were a notch filter 488/561/633 and one 594.

## Supporting Information Available

Deconvolution of the mass spectrum of the EITC–streptavidin conjugate (PDF)
