# Supplementary material for: Versatile Supramolecular Complex for Targeted Antimicrobial Photodynamic Inactivation
Source: Bioconjug Chem. 2022 Mar 10;33(4):666–76. doi: 10.1021/acs.bioconjchem.2c00067 (PMC9026257; doi:10.1021/acs.bioconjchem.2c00067)
Supplement: Supplementary file 1 — bc2c00067_si_001.pdf [file bc2c00067_si_001.pdf]

Supporting Information to

A versatile supramolecular complex for targeted antimicrobial photodynamic inactivation

Andrea Mussini[†,#], Eleonora Uriati[†,‡,#], Cormac Hally[†,§], Santi Nonell[§], Paolo Bianchini[‡], Alberto Diaspro[‡,&], Stefano Pongolini[£], Pietro Delcanale[†], Stefania Abbruzzetti[†,*], Cristiano Viappiani[†,*]

[†]Dipartimento di Scienze Matematiche, Fisiche e Informatiche, Università di Parma, Parco Area delle Scienze 7A, 43124, Parma, Italy

[‡]Nanoscopy @ Istituto Italiano di Tecnologia, via E. Melen 83B, 16152, Genova, Italy

[§]Institut Químic de Sarrià, Universitat Ramon Llull, Via Augusta 390, 08017, Barcelona, Spain

[&]DIFILAB, Dipartimento di Fisica, Università di Genova, via Dodecaneso 33, 16146, Genova, Italy

[£]Risk Analysis and Genomic Epidemiology, Istituto Zooprofilattico Sperimentale della Lombardia e dell’Emilia-Romagna, Strada dei Mercati, 13/A - 43126 Parma, Italy

[#]these authors contributed equally to this work

*Corresponding authors: stefania.abbruzzetti@unipr.it, cristiano.viappiani@unipr.it, Tel. +39 0521 905208

Mass spectrometry

Mass spectrometry on the full length proteins was carried out using an LTQ Orbitrap (Thermo Fisher Scientific) mass spectrometer. The proteins were separated in a Phenomenex Aeris™ PEPTIDE 3.6 µm XB-C18 (150 mm x 2.1 mm) reverse-phase column, developed in a 0.1% formic acid/water-0.1% formic acid/acetonitrile gradient (200 µl/min). The calculation of the AUC of the peaks was performed using the software Xcalibur™ (Thermo Fisher Scientific).

Elution of unlabelled strep through the reverse phase column showed a major peak at 12.56 min. Elution of EITC labelled strep at DOL<1 (meaning that some strep monomers will bear no EITC), resulted in two bands at 12.83 min. and 14.34 min., corresponding to unlabelled and labelled strep monomers, respectively. Deconvolution of mass spectrum of the peak of the native streptavidin monomers (elution time 12.83 min, **Figure S1** bottom) and of labeled streptavidin monomers (elution time 14.34 min, **Figure S1** top) evidences a mass difference of 704.7 Da, consistent with labeling of one EITC molecule per strep monomer.

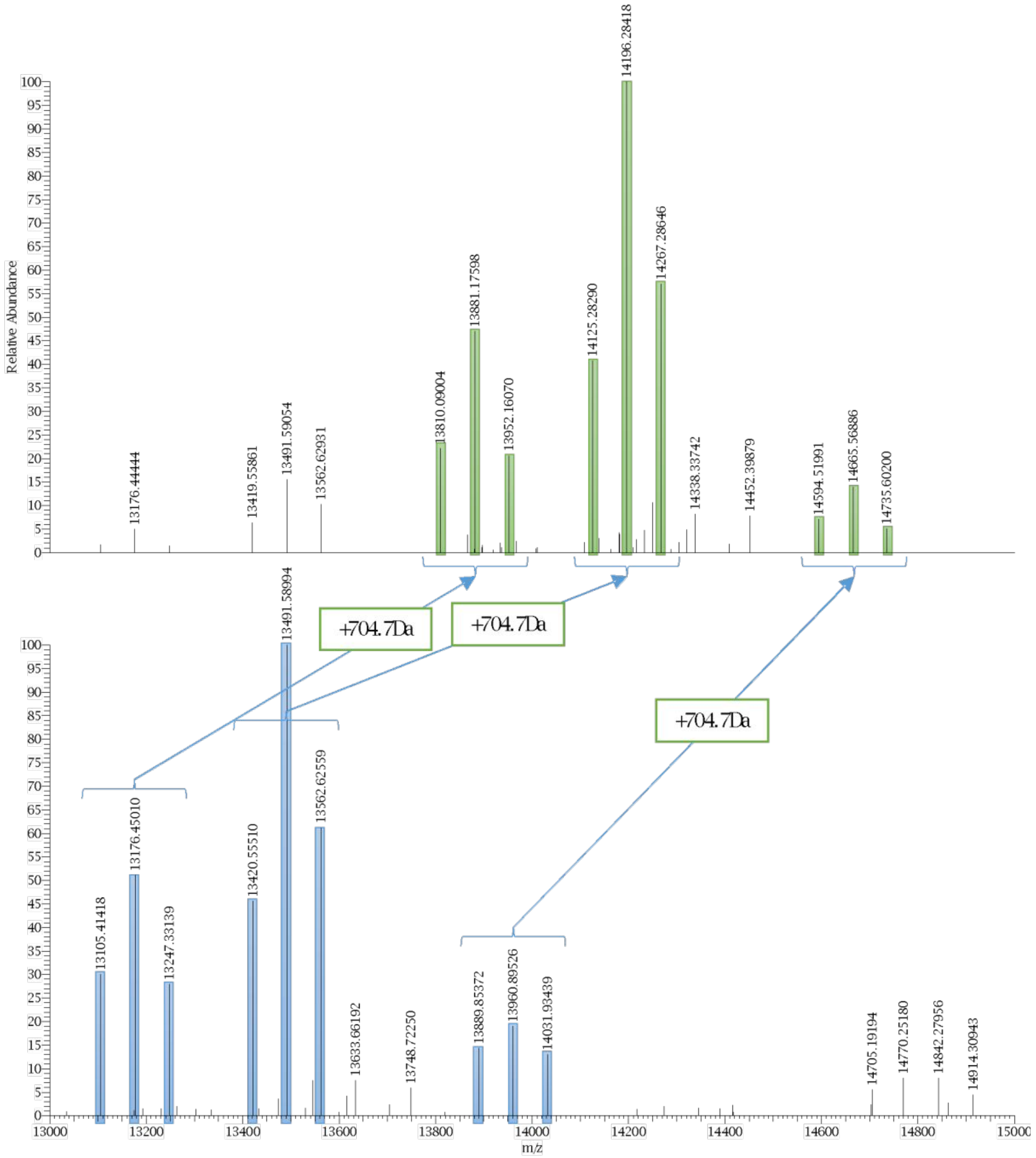

Figure S1. Deconvolution of mass spectrum.
